# Do We Need a World Health Insurance to Realise the Right to Health?

**DOI:** 10.1371/journal.pmed.0030530

**Published:** 2006-12-26

**Authors:** Gorik Ooms, Katharine Derderian, David Melody

## Abstract

There has been growing recognition in the international community that health should be considered a human right. Much less attention has been paid, however, to the ensuing legal obligation to provide international assistance.

There has been growing recognition in the international community that health should be considered a human right, a right enshrined by several international treaties. Much less attention has been paid, however, to the ensuing legal obligation to provide international assistance.

Kenneth Roth, director of Human Rights Watch, provided a rare comment endorsing the legal obligation to provide international assistance for health [1]. At the same time, he also underscored two problems associated with fulfilling this obligation: shared responsibility and progressive realisation (i.e., the obligation is not immediate but takes time to fulfil) [1].

The problem of progressive realisation lies in the acknowledgement that all economic, social, and cultural rights cannot be fully realised in a short period of time. This allows states to claim that they are doing or have done everything they can.

This problem has been partially addressed by the Committee on Economic, Social and Cultural Rights (“the Committee”), created to monitor states' achievements on the realisation of the rights recognised in the Covenant on Economic, Social and Cultural Rights (“the Covenant”) [2]. The Committee has stated that “a minimum core obligation to ensure the satisfaction of, at the very least, minimum essential levels of each of the rights is incumbent upon every State party” [3]. The Committee stressed that, in terms of the right to health, “a State party cannot, under any circumstances whatsoever, justify its non-compliance with the core obligations…which are non-derogeable” [4]. The realisation of the minimum essential level is understood to be immediate rather than progressive.

The obligation to provide assistance for health is further circumvented by a second problem: that of shared responsibility. Poor states can blame rich states for not honouring their obligation to provide assistance, thus leaving poor states with insufficient means to meet their core obligations. Rich states can blame poor states—and each other—for not doing enough.

A world health insurance could solve that problem by defining rights and duties for both rich and poor states. Wishful thinking? Maybe not. The basic requirements for a world health insurance have already been developed in theory and even in practice to a certain extent.

A world health insurance would shed different light on the problem of sustainability. Several effective health interventions are branded as “unsustainable” in poor countries, because national health budgets cannot afford them [5]. The option of substantially and permanently increasing national health budgets through international assistance is rarely considered. The creation of the Global Fund to Fight AIDS, Tuberculosis and Malaria (“the Global Fund”) demonstrates the merits of ambitious thinking: the provision of antiretroviral therapy (ART) to people living with AIDS, previously dismissed as unsustainable, became widely accepted as soon as the Global Fund provided a long-term funding perspective. Other health interventions deserve a similar approach.

## A Practical Framework for a World Health Insurance

The underlying principle of health insurance is the willingness to share health risks and the burden of health care. In national health insurance schemes, duty-bearing individuals pay a fair contribution; rights-holding individuals receive assistance in accordance with their health-care needs.

Transposed to a world health insurance, rich states would pay a fair contribution and poor states would have a right to assistance according to the health-care needs that they are unable to finance themselves. The creation of a world health insurance would therefore require: (1) A willingness to share health risks and the burden of health care between rich and poor states; (2) A mechanism to allocate resources to poor states; and (3) A mechanism to determine the contributions from rich states.

## Willingness to Share Health Risks and the Burden of Health Care

The principal obligation of the states that ratified the Covenant is “…to take steps, individually and through international assistance and cooperation, especially economic and technical, to the maximum of [their] available resources, with a view to achieving progressively the full realization of the rights recognized in the present Covenant” [6].

In its third general comment, the Committee concludes that “a minimum core obligation to ensure the satisfaction of, at the very least, minimum essential levels of each of the rights is incumbent upon every State party” [3]. In its 14th general comment, the Committee defines the core obligations regarding the right to health, including the obligation to “ensure the right of access to health facilities, goods and services on a non-discriminatory basis, especially for vulnerable or marginalized groups” and the obligation to “provide essential drugs, as from time to time defined under the WHO Action Programme on Essential Drugs” [4].

What if a poor state lacks the resources to provide such essential medicines and health care? The obligation to take steps to the maximum of its available resources refers both to the state's own resources and to those available from the international community [7]. The Committee has emphasised the duties of richer states: “For the avoidance of any doubt, it is particularly incumbent on State parties and other actors in a position to assist, to provide ‘international assistance and cooperation, especially economic and technical’ which enable developing countries to fulfil their core and other obligations…” [4].

Accordingly, rich states should provide the additional resources poor states need to provide a minimum level of health care—not as a matter of charity, but of meeting their legal obligations.

## A Mechanism to Allocate Resources to Poor States

The state remains the first duty-bearer toward its citizens. Poor states can only claim international assistance when they are unable to fulfil their obligations on their own.

The Commission on Macroeconomics and Health of the World Health Organization (WHO) estimates the minimum budget required to finance adequate levels of health care in poor countries to be US$35 per person per year [8]. This estimate is based on priority health interventions that do not exceed the core obligations as defined by the Committee [9]. Therefore, governments must spend at least US$35 per person per year on health care to fulfil their core obligations.

But governments have duties other than providing health care. How much should they allocate to health care to be able to say they have used the maximum of their available resources? We use the benchmark of 15% of total government expenditure adopted by African states in the 2001 “Abuja Declaration” [10]. In our exercise, if 15% of total government expenditure is insufficient, poor states can claim the remainder from rich states as Official Development Assistance (ODA) for health, through a world health insurance.

Table 1 estimates the remainders for 54 low-income countries, based on WHO information about national health accounts. Six low-income countries would not need additional ODA for health; for one (Somalia), the WHO lacks accurate information. The other 47 countries together would need about US$30 billion additional ODA for health annually.

**Table 1:**
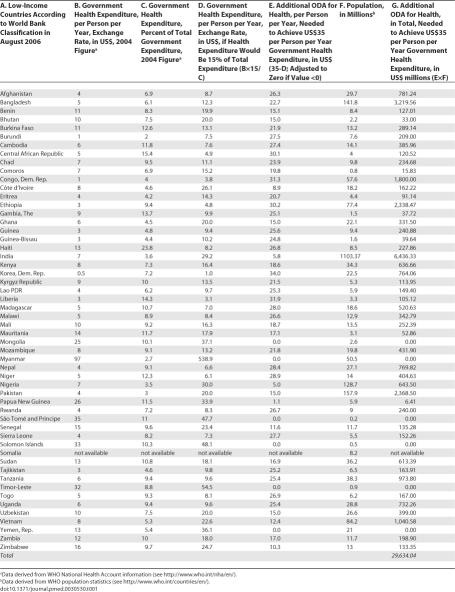
Low-Income Countries' Additional ODA for Health Needs to Achieve US$35 per Person per Year Government Health Expenditure

Claims for additional annual ODA for health of US$6 billion (India), US$3 billion (Bangladesh), or US$2 billion (Ethiopia) might seem grotesque. But without additional ODA, these countries simply cannot reach the expenditure level of US$35 per person per year, even with an allocation for health of 15% of their total budgets. Moreover, these figures are in line with the estimated additional ODA needed to meet all the Millennium Development Goals, including the health-related ones: US$48 billion per year by 2006 and US$74 billion by 2015 [11].

Currently, no single agency is equipped to deal with ODA claims of this magnitude. But if the international community is serious about the Millennium Development Goals, it must find innovative ways to increase ODA for health. The Global Fund has demonstrated that it is possible to increase ODA for health rapidly and in a transparent manner, based on country-owned proposals. The World Bank provides substantial funding to the health sectors of most low-income countries. Many of these countries have also developed common funding mechanisms for the health sector, in which several donors pool their contributions. A combination of these mechanisms would enable ODA for health to be increased substantially, rapidly, and in a transparent and reliable manner. In some countries, Global Fund grants are already integrated into the health sector common fund [12], and the World Bank and the Global Fund are working together to harmonise their grants [13]. The allocation of a substantial and open-ended increase of ODA for health is entirely feasible.

## A Mechanism to Determine the Contributions from Rich States

Once we know approximately how much poor states require to ensure the minimum essential level of the right to health, we need to determine which rich states should contribute, and how much.

The International Development Association (IDA)—the “soft loan arm” of the World Bank Group—has developed a burden-sharing mechanism based on adjusted gross national income [14]. The mechanism has no legal status, but is widely accepted as fair.

Table 2 lists 40 rich states contributing to the 14th replenishment of the IDA and estimates how they should share the burden of the additional ODA for health needed by the 47 poor states listed in Table 2.

**Table 2:**
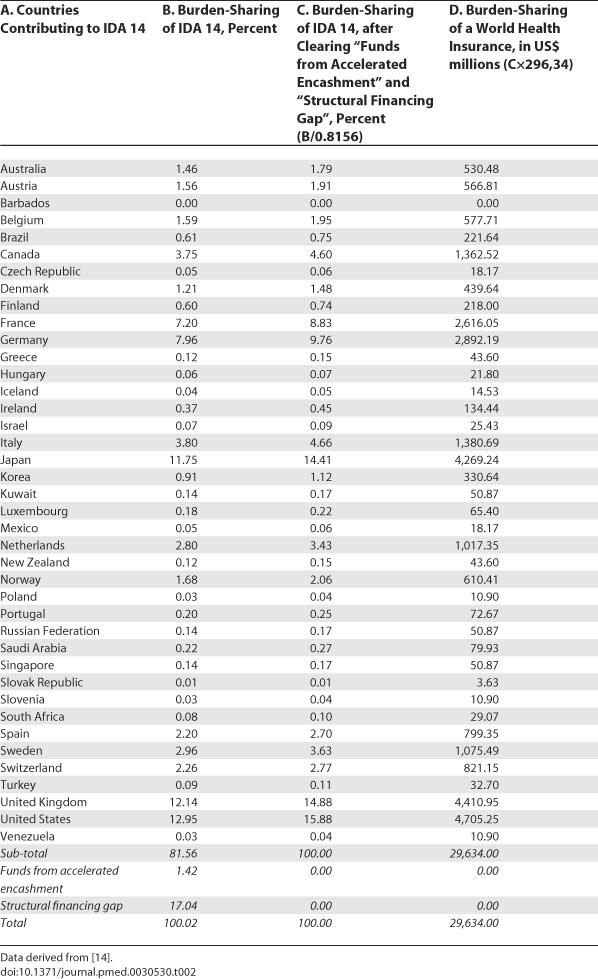
Burden Sharing of a World Health Insurance

Again, the bottom line is that burden sharing among rich states is a feasible way to provide additional ODA for health. We do not need to invent a world health insurance from scratch; we have all the basic requirements already.

## Do We Need a World Health Insurance?

The creation of the Global Fund revolutionised public health policies in low-income countries. Previously, the provision of ART in low-income countries was dismissed as unsustainable. After its creation, however, many low-income countries shifted their policies almost overnight, from a narrow focus on HIV prevention to a balanced mixture of prevention and treatment.

Was it the amount of available funding through the Global Fund that revolutionised policies to fight HIV/AIDS? Hardly. By May 2006, in just over three years, the Global Fund had disbursed US$2.26 billion [15]. This average of less than US$1 billion per year is a drop in the funding ocean compared with the overall amount of ODA of almost US$100 billion in 2003 [16]. Even if compared with ODA for health (almost US$7 billion in 2003), Global Fund funding represents an increase of less than 15%. Why did it make such a difference?

The answer may lie in the problem of sustainability. The Global Fund recently closed its sixth call for proposals. The guidelines mention:

“The applicant should describe how grant-supported activities and interventions will…help to establish and build sustainable systems (including management and financial systems); human resource capacity; technical competence; and other foundations to support the continuity of planned interventions beyond the program term, as appropriate” [17].

In other words, the Global Fund wants every element of the intervention to be sustainable, except the funding which it ensures itself.

The Global Fund's new understanding of sustainability—relying on sustained international assistance, not on present or future selffinancing—seems to have influenced the thinking of rich states. The Political Declaration on HIV/AIDS adopted by the United Nations General Assembly in June 2006 commits to the “provision of funds [for ART] in a sustained manner” [18]. This is fundamentally different from the earlier commitment to “provide [ART] progressively and in a sustainable manner”, made in the “Declaration of Commitment” following the United Nations General Assembly Special Session on AIDS in June 2001 [19].

The reliability of Global Fund funding also helps to overcome other obstacles. For decades the World Bank and the International Monetary Fund (IMF) have imposed ceilings on public health expenditure [20]. Low-income countries are not allowed to “break” the ceiling, or to increase their health budgets beyond the ceiling, even if they obtain additional ODA to cover additional expenses. Organisations like the WHO have maintained that “Financial ceilings…may need to be stretched” [21], but the position of the World Bank is clear: “[I]t is not prudent for countries to commit to permanent expenditures for such items as salaries for nurses and doctors on the basis of uncertain financing flows from development assistance funds” [22]. The World Bank and the IMF seem willing to make an exception for additional ODA coming from the Global Fund, however, because it is reliable and predictable. In Malawi, for example, the government and the IMF agreed that “[t]he ceiling on central government wages and salaries will be adjusted upward (downward) by the full amount of donor-funded supplementary wages and salaries for the health sector that is greater (less) than the program baseline” [23].

Improving the reliability of ODA for health would also improve the overall impact of existing ODA flows. For a low-income country it is easier to obtain ODA to build a hospital, or technical assistance to learn how to run that hospital, than for “recurrent costs” like the salaries of the medical staff or the medicines needed to run the hospital.

**Figure 1:**
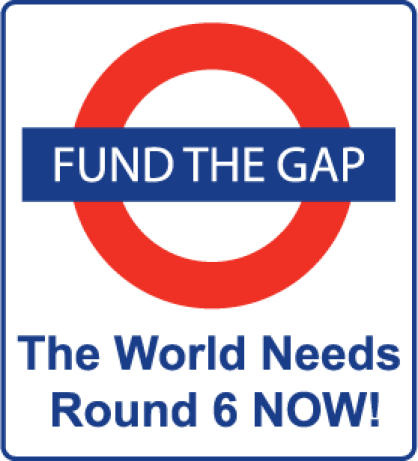
Fund the Gap A worldwide civil society campaign to save round six of the Global Fund.

If the Global Fund is to deliver on its promise of providing reliable and predictable funding, it will need reliable and predictable contributions itself. A burden-sharing mechanism is therefore essential. Supporters of the Global Fund have been demanding such a mechanism since its creation [24], as indeed has the Global Fund itself [25]. It cannot continue to rely on civil society campaigns to make sure it receives the resources needed to the approved proposals it calls for, as it has in 2006 (Figure 1).

Although the Global Fund is limited to three diseases, AIDS, tuberculosis, and malaria are not the only diseases that deserve more ambitious interventions. High levels of infant mortality caused by severe acute malnutrition, for example, have long been a cause of fatality in low-income countries. The provision of milk paste is effective, but its price remains an obstacle [26]. Providing milk paste in poor health systems, or on poor health budgets, will only be possible if traditional notions of sustainability are rejected in favour of that of sustained funding. Mindsets still need to change at all levels—even that of the Executive Director of UNICEF, who continues to insist that effective interventions to reduce infant mortality should be “phased in according to the ability of both the health system to deliver them at scale, and of governments to afford them and to sustain them in the longer term” [27].

## A Lesson for Health Nongovernmental Organisations

The creation of a world health insurance is a matter for governments, not for nongovernmental organisations (NGOs). But the lesson for NGOs is that a world health insurance, or any similar arrangement to increase ODA for health in a reliable and predictable manner, does not need to be invented from scratch.

Our view sheds a different light on the traditional notions of sustainability that inform many NGOs' health-care interventions—that recurrent costs should be covered locally and that governments should be able to sustain them in the longer term. There is no international law stipulating that ODA should be limited in time, or that local resources should cover recurrent costs. On the contrary, international law requires that rich states assist poor states to meet the minimum essential level of the right to health.

NGOs could advocate for the creation of a world health insurance while continuing to align their interventions with traditional ideas about sustainability. Or they could act as if a world health insurance existed already and focus on delivering effective health interventions. We would argue that it was the provision of ART in low-income countries that forced rich and poor states to find a solution for the sustained financing of ART, not the other way around. Other effective health interventions deserve a similar strategy.

## Abbreviations

ART: antiretroviral therapy
IDA: International Development Association
IMF: International Monetary Fund
NGO: nongovernmental organisation
ODA: Official Development Assistance
WHO: World Health Organization
